# Erupted Compound Odontomas: A Case Report

**DOI:** 10.5681/joddd.2011.007

**Published:** 2011-03-18

**Authors:** Avinash Tejasvi M.L., Balaji Babu B

**Affiliations:** ^1^ M.D.S, Senior Lecturer, Department of Oral Medicine and Radiology, Kamineni Institute of Dental Sciences, Narketpally, Andhra Pradesh, India; ^2^ M.D.S, Reader, Department of Oral Medicine and Radiology, Kamineni Institute of Dental Sciences, Narketpally, Andhra Pradesh, India

**Keywords:** Compound odontoma, compound complex odontoma, erupted odontoma, hamartoma

## Abstract

The tumors in which odontogenic differentiation is fully expressed are the odontomas. Odontomas are considered as hamartomas rather than a true neoplasm. These tumors are composed of enamel, dentine, cementum and pulp tissue. It is most commonly associated with the eruption of the teeth. They are usually discovered on routine radiographic examination. In exceptional cases, the odontoma erupts in to the mouth. Nine cases of erupted compound odontomas are reported in the English literature, and the present paper reports another case of an erupted compound odontoma in a 22-year-old female patient.

## Introduction

Odontomas erupting into the oral cavity are extremely uncommon, with the first case being reported in 1980 by Rumel et al.^1^ The term “odontoma” by definition alone, refers to any tumor of odontogenic origin.^2^ They are considered as developmental anomalies rather than a true neoplasm.Paul Broca was the first person who coined the name odontoma in 1867.^3^ Broca defined the term as tumors formed by the overgrowth or transitory of complete dental tissue.^3^ Odontomas are benign tumors of odontogenic origin combining mesenchymal and epithelial dental elements.^4^ The etiology of odontoma has been attributed to various pathological conditions like local trauma, inflammatory and or infectious processes, hereditary anomalies (Gardner’s syndrome, Hermann’s syndrome). Odontoblastic hyperactivity and alterations in genetic component is responsible for controlling dental development. Persistence of a portion of lamina may be an important factor in the etiology of a compound odontoma.^5^Most odontomas are asymptomatic; occasionally, however, signs and symptoms relating to their presence do occur.Histologically, they are composed of different dental tissues, including enamel, dentine, and cementum and, in some cases, pulp tissue.^4^

According to world health organization (WHO) classification, odontomas are classified as complex odontoma and compound odontomas. A malformation in which all dental tissues are formed, but occurring in less orderly pattern is complex odontoma. A malformation in which all dental tissues are arranged in a more orderly pattern than complex odontoma is compound odontoma.^2^ Howards lists odontoma as fourth category of supernumerary teeth.^6^ However, this classification is universally not accepted. Although there are many reported cases of odontomas in the literature, erupting odontoma is a rare occurrence. In this paper, a case of an erupted compound odontoma in a young female is reported.

## Case Report

**Figure 1:**
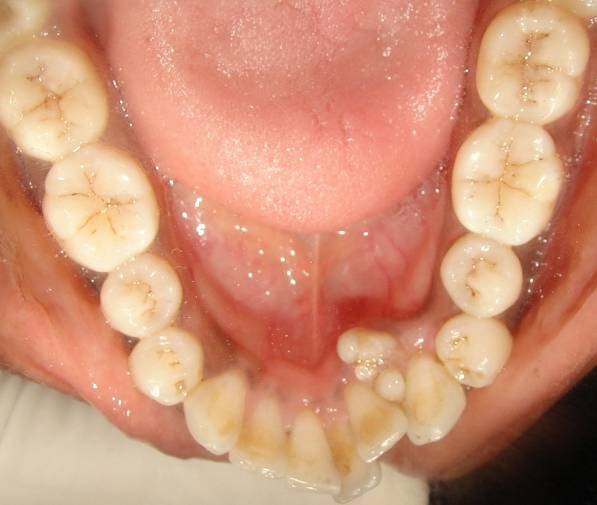
Intraoral view showing erupted odontomas between lateral incisor and canine in the third quadrant.

A 22-year-old female who was apparently healthy reported to the Department of Oral Medicine and Radiology with the chief complaint of forwardly placed upper and lower front teeth. Medical history was non contributory and there were no hereditary disease in antecedents. Extra-oral examination was unremarkable. Clinical intraoral examination revealed normal compliment of teeth in upper and lower arch with multiple yellowish-brown, dental tissue-like lesion which had a calcified appearance with an irregular surface in the third quadrant. It was situated on the lingual aspect in between the lower left lateral incisor and the lower left canine, which was erupting into the oral cavity (Figure 1). Lesions were hard in consistency, tooth-colored and measured about 5-6 mm in diameter. It had a calcified appearance and an irregular surface which was suggestive of small multiple teeth like structures. There was no inflammation, pain, infection, erythema, or ulceration of the floor of the mouth or tongue. On palpation, these multiple teeth-like structures were asymptomatic and were not mobile.

**Figure 2:**
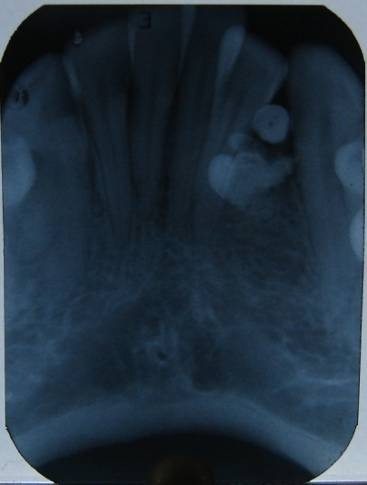
Intraoral periapical radiograph showing a radiopaque lesion compatible with compound odontoma between lower left lateral incisor and canine, which has displaced the disto-inferior region of the lateral incisor.

**Figure 3:**
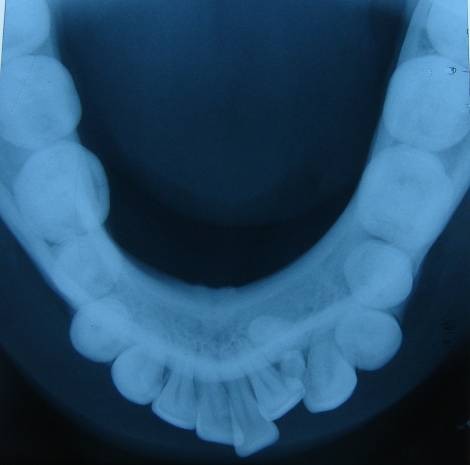
Mandibular cross-sectional occlusal radiograph showing compound odontomas which are well-defined with smooth borders. Contents of these lesions are radiopaque, appearing like a number of denticles or tooth-like structures.

**Figure 4:**
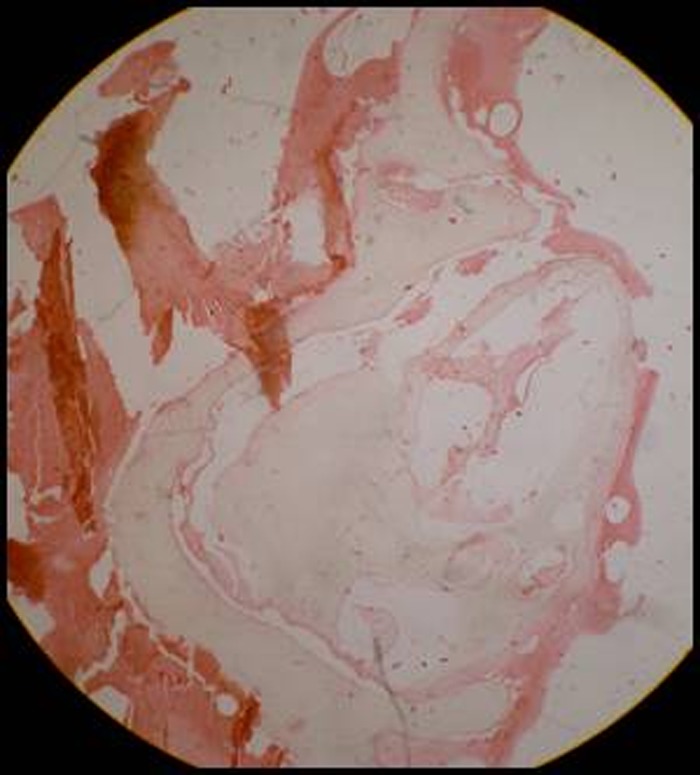
Photomicrograph showing decalcified dentin, decalcified enamel intermixed with haphazardly arranged areas containing remnants of immature enamel matrix suggestive of compound odontoma.

Patient was advised with a set of radiographs including intraoral periapical and occlusal radiographs to assess the precise location and extent of the lesion, as well as its relations to the surrounding anatomical structures (Figures 2 & 3). Multiple small teeth-like radiopaque structures were seen in lower anterior mandible and were not associated with any unerupted teeth. They were well defined with smooth borders and contents of the lesions were radiopaque which were appearing like number of denticles or tooth-like structures. These radiopaque structures were present between lower left lateral and canine. These denticles displaced the disto-inferior region of the lateral incisor which led to crowding in lower anterior teeth. Considering the clinical and radiographic presentations, a radiographic diagnosis of erupted compound odontomas was determined. Under local anesthesia the access to the mass was achieved through an intraoral approach and odontomas were extracted and subjected to histopathological examination which confirmed the diagnosis of compound odontoma (Figure 4).

## Discussion

The term odontoma was first used by Paul Broca in 1867. Later, In 1914 Gabell, James and Pyne grouped odontoma according to their developmental origin as epithelial, composite (epithelial and mesodermal) and connective tissue. In 1946, Thoma & Goldman formulated a classification for odontomas.^7^ Eruption of odontomas is infrequent in the literature.

The first case of an erupted odontoma was described in 1980 by Rumel et al.^6^ Later in 2009, Gabriel Serra-Serraet al^8^ reported in stating that, since 1980 only 17 cases of erupted odontomas were documented in the literature, and including their 3 patients, it would be 20 cases in the literature. Of the 20 reported cases of erupted odontoma, 9 correspond to compound odontomas and 11 to complex odontomas. The case in discussion here is the 21^st^ erupted odontomas case and the 10^th^ case of erupted compound odontoma reported in the English literature. A case of erupting multiple odontoma is also reported in the literature.^9^

Odontomas are benign tumors containing various tissue components of the teeth. They are most common odontogenic tumors constituting 22% of all odontogenic tumors of the jaws.^10^ Pain and swelling are the most common symptoms when odontomas erupt, followed by malocclusion. Recurrent infection following eruption into the oral cavity has been reported but the patient was asymptomatic in this case.^11^ There are two types of odontomas: complex odontomas and compound odontomas – the latter being twice as frequent as the former. Compound odontomas show a predilection in the anterior section of the upper maxilla while complex odontomas are typically found in the posterior mandibular region.^12^ In the presented case the erupted odontoma was the compound type and it presented on the mandibular anterior region, which is a rare occurrence.

Radiographically, odontoma presents as a well-defined radiopacity situated in bone but with a density that is greater than bone and equal to or greater than that of a tooth. It contains foci of variable density. It is present with a radiolucent halo, typically surrounded by a thin sclerotic line, surrounding the radiopacity. Radiolucent zone is the connective tissue capsule of a normal tooth follicle. Thin sclerotic line resembles the corticated border seen in a normal tooth crypt. Developmental stages can be identified based on radiologic features and the degree of calcification of the lesion at the time of diagnosis.^13^ First stage is characterized by radiolucency due to the absence of dental tissue calcification, the second or intermediate stage shows partial calcification and the third or classically radiopaque stage exhibits predominant tissue calcification with the surrounding radiolucent halo described above.^14^ In the present reported case, the radiographic appearance was seen as a multiple dense radiopaque structure outside the jaw bone, with clear external margins presenting normal organization of dental tissues, i.e. enamel, dentine, and pulp. Treatment of choice is surgical removal of the lesion in all cases, followed by histopathological study to confirm the diagnosis. In this case, surgical removal of the lesion was performed. The excised specimen was subjected to histopathologic examination, the report of which matched that of radiographic diagnosis.

## Conclusion

A rare case of erupted compound odontomas has been reported. The most important and interesting feature in this case was that the odontomas were erupted from the lower anterior region of the jaw into the oral cavity like a tooth, which is a very rare occurrence, causing a mild disturbance to the adjacent teeth. A surgical intervention was done and the odontomas were extracted and further orthodontic treatment was followed for the patient.
